# Unusual Lower Critical Solution Temperature Phase Behavior of Poly(benzyl methacrylate) in a Pyrrolidinium-Based Ionic Liquid

**DOI:** 10.3390/molecules26164850

**Published:** 2021-08-11

**Authors:** Brian R. Carrick, Claire L. Seitzinger, Timothy P. Lodge

**Affiliations:** 1 Department of Chemistry, University of Minnesota, Minneapolis, MN 55455, USA; carri373@umn.edu (B.R.C.); seitz133@umn.edu (C.L.S.); 2 Department of Chemical Engineering and Materials Science, University of Minnesota, Minneapolis, MN 55455, USA

**Keywords:** ionic liquid, polymers, poly(benzyl methacrylate), LCST, phase diagram

## Abstract

Polymer/ionic liquid systems are being increasingly explored, yet those exhibiting lower critical solution temperature (LCST) phase behavior remain poorly understood. Poly(benzyl methacrylate) in certain ionic liquids constitute unusual LCST systems, in that the second virial coefficient (*A*_2_) in dilute solutions has recently been shown to be positive, indicative of good solvent behavior, even above phase separation temperatures, where *A*_2_ < 0 is expected. In this work, we describe the LCST phase behavior of poly(benzyl methacrylate) in 1-butyl-1-methylpyrrolidinium bis(trifluoromethylsulfonyl)imide for three different molecular weights (32, 63, and 76 kg/mol) in concentrated solutions (5–40% by weight). Turbidimetry measurements reveal a strong concentration dependence to the phase boundaries, yet the molecular weight is shown to have no influence. The critical compositions of these systems are not accessed, and must therefore lie above 40 wt% polymer, far from the values (ca. 10%) anticipated by Flory-Huggins theory. The proximity of the experimental cloud point to the coexistence curve (binodal) and the thermo-reversibility of the phase transitions, are also confirmed at various heating and cooling rates.

## 1. Introduction

Ionic liquids (ILs) are an important class of “designer solvents”, able to access numerous desired physical properties through modest modifications of their chemical structure. ILs offer thermal, chemical, and electrochemical stability, as well as high ion conductivity and negligible volatility, thereby showing great promise as greener alternatives to traditional solvents [1,2,3,4,5]. Incorporating a polymer matrix into these ILs imparts structural and mechanical integrity to the resulting material, while retaining the liquid-like diffusivity of the IL [6,7,8]. This has enabled their use for separation membranes, printable electronics, battery electrolytes, and self-healing materials [9,10,11,12,13,14,15]. However, this growing class of smart materials poses some interesting fundamental questions.

Polymer/IL systems exhibiting lower critical solution temperature (LCST) phase behavior remain poorly understood, as their behavior is not anticipated by the classical Flory-Huggins theory [16,17]. LCST behavior is often attributed to the formation of well-oriented solvation structures surrounding the polymer, causing these solutions to be homogenous at low temperatures but to undergo liquid-liquid phase separation upon heating [18,19,20,21,22,23,24,25,26,27,28,29,30,31]. Watanabe and co-workers have demonstrated that the LCST behavior of polyethers in imidazolium-based ionic liquids is due to hydrogen bonding between the C2 hydrogen of the imidazolium cation and the polymer oxygen; this conclusion is supported by computational studies by Costa and Ribeiro [18,19,22,23]. Lee et al. have expanded upon this work by constructing a complete temperature-composition phase diagram for poly(ethylene oxide) (PEO) in 1-ethyl-3-methylimidazolium tetrafluoroborate ([EMIM][BF_4_]) via turbidity and small-angle neutron scattering measurements [20,21]. They also explored the LCST phase behavior of poly(n-butyl methacrylate) (PnBMA) in 1-alkyl-3-methylimidazolium bis(trifluoromethylsulfonyl)imide ([C_n_MIM][TFSI]); they proposed that the IL organizes around solvatophobic n-butyl groups, analogous to the cage-like structure formed in aqueous poly(*N*-isopropylacrylamide) solutions [24,25,32].

A system of particular interest is poly(benzyl methacrylate) (PBzMA) in ILs. Watanabe and co-workers first observed its LCST behavior and proposed that the formation of clathrates via cation-π interactions between imidazolium cations and benzyl groups leads to entropic stabilization during mixing [26,27,28,29]. Later work also observed intramolecular π-π stacking between adjacent benzyl groups, creating compact polymer aggregates rather than flexible random coils, as is typically observed within good solvents [17,30]. Recently, Kharel et al. determined the second virial coefficient to be consistently positive for PBzMA in dilute imidazolium- and pyrrolidinium-based ionic liquid solutions, indicating the ILs are reasonably good solvents, even above temperatures wherein phase separation occurs at higher concentrations. Thus, the theta temperature determined in very dilute solutions is well *above* the separation temperature of these systems, an unusual characteristic [31]. The critical concentration of several PBzMA/IL systems have also been observed to be significantly shifted toward polymer-rich compositions, contrary to the solvent-rich compositions anticipated by Flory-Huggins theory [16,31,33,34].

**Figure 1:**
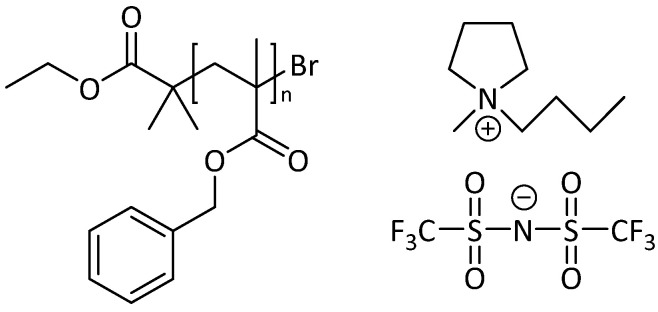
Chemical structure of poly(benzyl methacrylate) (PBzMA, left) and 1-butyl-1-methylpyrrolidinium bis(trifluoromethylsulfonyl)imide ([BMP][TFSI], right).

Few phase diagrams for polymers in ILs have been explored over wide composition ranges, and, to our knowledge, no polymer system involving pyrrolidinium-based ILs has been fully characterized. These non-aromatic ILs have been shown to be less toxic than their aromatic counterparts; thus, they can potentially be used as safer alternatives for applications once there is a better understanding of their behavior in polymer solutions [35]. In this work, we expand upon the previous studies by exploring the phase behavior of PBzMA in 1-butyl-1-methylpyrrolidinium bis(trifluoromethylsulfonyl)imide ([BMP][TFSI]) for a wide range of concentrations via turbidimetry as a function of polymer molecular weight (Figure 1). Additionally, the thermal reversibility of the phase transition is examined and discussed.

## 2. Results

At low temperatures, PBzMA is completely miscible with [BMP][TFSI] and a clear solution is observed. As can be seen in Figure S3, upon heating, a sharp drop in transmittance occurs during phase separation as the solution becomes cloudy; the temperature at which this transpires is the cloud point (*T*_CP_). The subsequent increase in transmittance at even higher temperatures is due to the formation of distinct phases. When annealed at temperatures above *T*_CP_ for several days, the formation of clear coexisting layers is achieved. The lower and upper phases consist of a [BMP][TFSI]-rich liquid and a PBzMA-rich viscoelastic liquid, respectively, as the density of [BMP][TFSI] (≈1.40 g/cm^3^) is higher than that of PBzMA (≈1.18 g/cm^3^). However, if these distinct layers have not fully developed before the solution is cooled to room temperature, the top layer remains opaque for months when stored in a vacuum desiccator. This suggests that the PBzMA-rich phase is metastable under these conditions, presumably due to kinetic limitations (e.g., reflecting chain entanglement and/or the glass transition).

**Figure 2:**
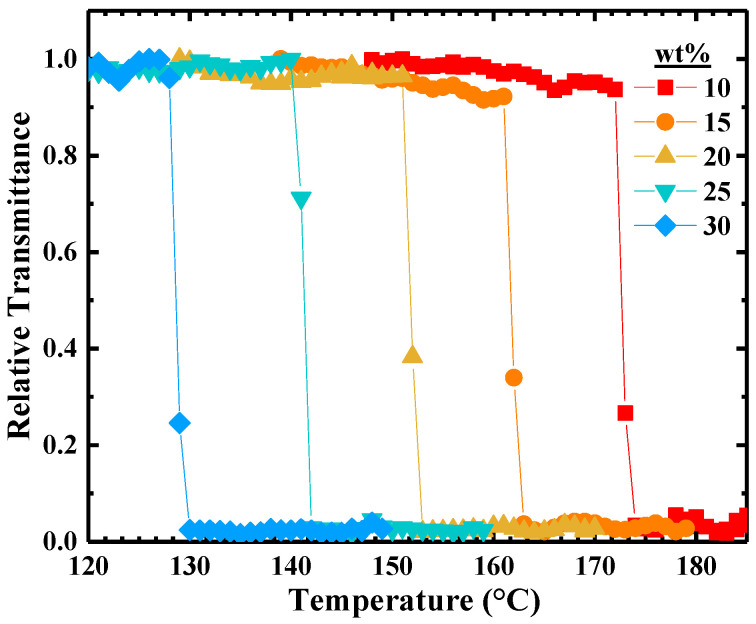
Temperature dependence of relative transmittance for PBzMA-63 in [BMP][TFSI] measured at a heating rate of 1 °C/min. The concentration of PBzMA in solution is indicated in wt%. The solid lines serve as guides to the eye.

The concentration dependence of *T*_CP_ was explored for PBzMA-63 in [BMP][TFSI] and is illustrated in Figure 2, where the relative transmittance of each solution is plotted as a function of temperature on heating. The relative transmittance rapidly decreases (within seconds) upon crossing *T*_CP_, irrespective of concentration. Accordingly, *T*_CP_ for the 10, 15, 20, 25, and 30 wt% PBzMA-63 solutions were determined to be 174, 162, 152, 141, and 129 °C, respectively. In comparison, previous work by Kharel et al. reported that the cloud point temperatures of 71 kDa PBzMA in 1-butyl-3-methylimidazolium bis(trifluoromethylsulfonyl)imide ([BMIM][TFSI]), the imidazolium-based IL counterpart to [BMP][TFSI], decrease from 154 to 143 °C as the concentration increases from 10 to 20 wt% [31]. These temperatures are modestly lower than the corresponding pyrrolidinium-based IL solutions, thereby suggesting a slightly increased miscibility with [BMP][TFSI] compared to [BMIM][TFSI].

**Figure 3:**
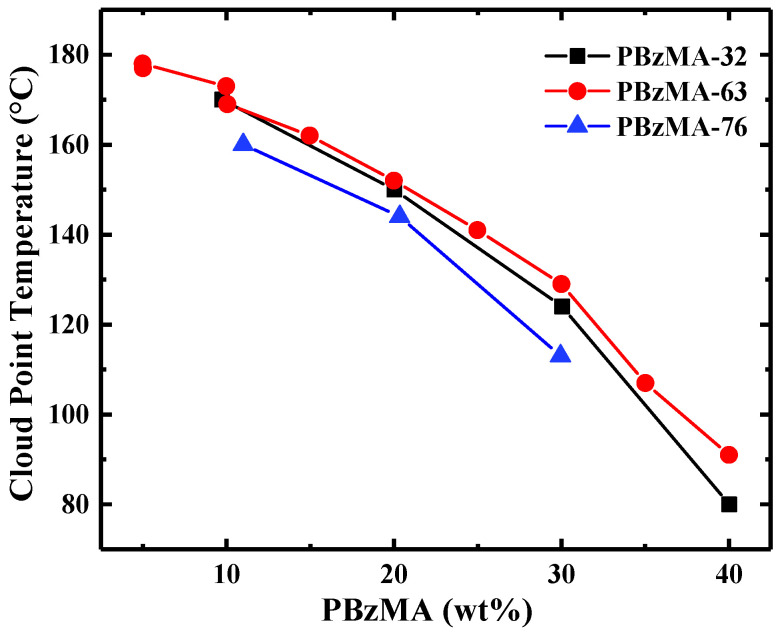
Composition dependence of the cloud point temperature for PBzMA in [BMP][TFSI] for different molecular weights measured at a heating rate of 1 °C/min. The molecular weights are PBzMA-32 (black squares), PBzMA-63 (red circles), and PBzMA-76 (blue triangles). The solid lines serve as guides to the eye.

An experimentally observed cloud point generally lies between the binodal (coexistence) and spinodal (stability limit) curves. For polymer solutions, *T*_CP_ determined via slow temperature variation often serves as a good approximation for the binodal curve (where distinct phases co-exist in equilibrium), as the rate of phase separation is dependent on nucleation of the phases, plus possibly the disassociation kinetics of the solvation structure. This assignment will be confirmed subsequently. The temperature-composition phase diagram in Figure 3 shows the cloud points, as determined by transmittance measurements exemplified in Figure 2, for three PBzMA molecular weights as a function of polymer concentration. The cloud point decreases monotonically with increasing concentration, with no suggestion of an approaching minimum. Flory–Huggins theory predicts that the critical composition, *Φ*_c_, (the composition corresponding to the lowest *T*_CP_) should be shifted toward more dilute concentrations with increasing chain length (*Φ*_c_ ≈ *N*^−1/2^, where *N* is the volumetric degree of polymerization with respect to a solvent molecule). However, it is observed that *T*_CP_ is apparently independent of molecular weight, and that it is still decreasing as the concentration of polymer increases to 30/40 wt% PBzMA, indicating that *Φ*_c_ lies within the polymer-rich regime of the phase diagram, far above the expected values.

**Figure 4:**
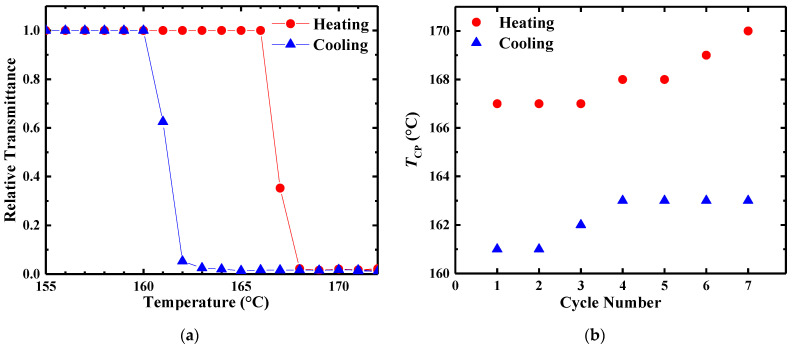
(**a**) Temperature dependence of relative transmittance at 628.3 nm for 10 wt% PBzMA-63 upon heating and cooling at a rate of ±1 °C/min. (**b**) Cycle dependence of the cloud point temperature (*T*_CP_) upon heating and cooling at a rate of ±1 °C/min for seven thermal cycles.

The reversibility of the phase transition indicated by the experimental cloud point was then explored by heating a 10 wt% PBzMA-63 in [BMP][TFSI] solution from 23 to 172 °C, and cooling from 172 to 23 °C, at a rate of ±1 °C/min for seven cycles. The solution was not allowed to anneal above *T*_CP_, thereby inhibiting the formation of individual layers. The temperature-dependent transmittance curve for the first cycle is shown in Figure 4a, where *T*_CP_ upon heating and cooling are 167 and 161 °C, respectively, demonstrating that the mixture persists after phase separation until cooled a few degrees below *T*_CP_. This narrow hysteresis upon slow temperature variation (≈6 °C) confirms that the cloud point is a good approximation for the binodal temperature. It can also be seen that *T*_CP_ increases modestly from 167 to 170 °C upon heating, and from 161 to 163 °C upon cooling, as the cycle number increases (Figure 4b); the hysteresis remains approximately constant.

**Figure 5:**
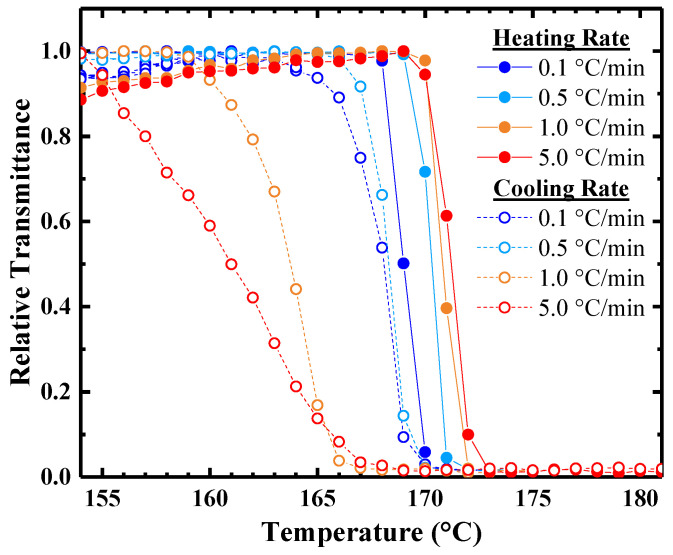
Temperature dependence of relative transmittance at 628.3 nm for 10 wt% PBzMA-32 in [BMP][TFSI] at heating and cooling rates of ±0.1, ±0.5, ±1.0, and ±5.0 °C/min.

To probe the heating/cooling rate sensitivity of the phase separation, the relative transmittance of 10 wt% PBzMA-32 in [BMP][TFSI] solutions was measured as a function of temperature for heating and cooling rates of 0.1, 0.5, 1.0, and 5.0 °C/min (Figure 5). The tight grouping of the *T*_CP_ values and the uniform sharp drop in transmittance upon reaching *T*_CP_ for the heating rates under study further confirms that *T*_CP_ upon heating lies close to the binodal curve; a factor of 50 increase in heating rate only changes *T*_CP_ by 2 °C. On the other hand, the thermal hysteresis widens from 1 to 14 °C as the cooling ramp rate increases from 0.1 to 5.0 °C/min. This is quite reasonable, as re-mixing typically requires finite time.

## 3. Discussion

Polymer solutions exhibiting strong and directional interactions, such as hydrogen bonding, can exhibit LCST phase behavior, and therefore do not conform to the assumptions of the basic Flory-Huggins theory (i.e., only weak, dispersive interactions). Many aqueous polymer systems have an LCST, due to the H-bonding network of water, and the tendency to form cage-like solvation structures. However, aqueous applications that exploit LCST behavior are limited to relatively low temperature windows [36,37]. Polymers in ILs are an emerging class of solutions that can exhibit this interesting phase behavior, presumably due to strong interactions that structure the solvent ions around the polymeric solute. In general, ILs show great promise due to their thermal stability, negligible vapor pressure, and tunable physico-chemical properties. As previously mentioned, small changes in the IL or polymer structure can cause large differences in the behavior of such systems. For example, *T*_CP_ for PBzMA in [BMP][TFSI] exhibits a stronger concentration dependence in comparison to other LCST systems such as PEO in [EMIM][BF_4_], PnBMA in [BMIM][TFSI], and even PBzMA in [BMIM][TFSI] [20,25,31]. We hypothesize that this dependence arises from interpolymer interactions between benzyl groups playing a pivotal role during phase separation. Previous work by Fujii et al. demonstrated that there are no significant interpolymer interactions between PBzMA chains in [EMIM][TFSI] at room temperature (below *T_CP_*) [30]. However, as the cation-π clathrate de-solvates during phase separation, benzyl groups from different chains begin to overlap as segments aggregate. By increasing the concentration of PBzMA, this stabilization becomes more prevalent and drives phase separation to lower temperatures by promoting the formation of solvent-rich and polymer-rich domains, favored by cation-π and π-π interactions, respectively.

In contrast to Flory-Huggins theory, the critical composition of PBzMA in [BMP][TFSI] appears to be shifted to the polymer-rich regime of the phase diagram. A similar shift has also been observed in PEO/[EMIM][BF_4_] solutions, with critical compositions varying from 50 to 80 wt% polymer [20,21]. The origin of this unusual phenomenon remains to be fully elucidated, but several mechanisms have been considered. White and Lipson hypothesize these shifts to be caused by a stronger cohesive energy density in the IL, and possible aggregation of the IL within polymer-rich mixtures [38]. However, these criteria should also be satisfied by poly(ethyl glycidyl ether) in [C_n_MIM][TFSI], whereas Watanabe and co-workers have revealed that the critical composition of this system is shifted toward dilute concentrations [18,19]. Another explanation, due to de Gennes, posits the formation of a dense polymer phase in equilibrium with the bulk solution due to inter-chain associations [39]. This could serve as a plausible explanation for PBzMA/IL solutions, as π-π stacking between benzyl groups would facilitate the formation of this phase, but recent light scattering studies have shown that PBzMA does not aggregate prior to phase separation in dilute solutions [31]. A third possibility presented by Schafer-Soenen and co-workers is that the interaction parameter is strongly dependent on the solution concentration [40]. This looks to be a promising path, as recent work from our group has already observed unusual behavior with PBzMA and ILs, in that in very dilute solutions, the second virial coefficient is positive, indicating good solvent behavior, at temperatures that exceed *T*_cp_ at higher concentrations [31]. An increase in the interaction parameter with concentration could resolve this apparent contradiction, as this would result in a decrease in miscibility with higher concentrations. It would also imply that the second virial coefficient, which is inevitably measured in very dilute solutions, is not a reliable indicator of the phase boundary. Note that the independence of *T*_CP_ on molecular weight for PBzMA in [BMP][TFSI] at high molecular weights (Figure 3) is not unusual for LCST systems. For example, the phase separation temperatures of PEO in [EMIM][BF_4_] becomes independent of *N* above 20 kDa [21]. Within the context of Flory–Huggins theory, this result is a natural consequence of a negative enthalpy of mixing combined with a substantial unfavorable entropy of mixing [17].

In general, liquid–liquid phase separation in the metastable region of the phase diagram proceeds by nucleation and subsequent growth of droplets of the minor phase (in this case, the polymer-rich phase). The alternative is spinodal decomposition, but the near reversibility of the experimental cloud point, with significant hysteresis, suggests a nucleation and growth process. Cloud point values upon heating and cooling typically do not coincide, as phase separation and remixing are kinetic processes that occur from different initial conditions. However, the concentrations are quite high in this system, and therefore the possibility of becoming trapped in a very dilute, metastable one-phase state is correspondingly low. For PBzMA/[BMP][TFSI] solutions, the visible onset of phase separation upon heating is rapid, typically occurring within seconds upon crossing *T*_CP_. These results suggest rapid dissociation of the cation-π clathrate and aggregation of PBzMA segments, both of which are molecular scale processes that can occur on much shorter time scales than the accessible heating rates. On the other hand, re-solvation and re-mixing upon cooling are limited by the mutual diffusion of PBzMA and solvent. This becomes readily apparent when examining the reversibility of the phase transition. As samples are annealed above *T*_CP_, the formation of two phases is thermodynamically favored and PBzMA migrates preferentially toward the top of the vial (Figure S3). If the system is cooled below *T*_CP_ before distinct layers are formed, the polymer chains become re-solvated and begin to diffuse into the solvent-rich lower domains. The apparent increase in *T*_CP_ as the cycle number increases (Figure 4b) suggests that the local polymer concentration within the path of the beam decreased. Without stirring, the change in local concentration after 10 min spent above *T*_CP_ is not completely reversed after almost 5 h spent below *T*_CP_, indicating that remixing is mass-transport-limited. This is also exemplified by the widening of the cloud point hysteresis (Figure 5) as the ramp rate increases. Thus, caution needs to be taken when handling solutions near *T*_CP_ as their compositions are rapidly altered during phase separation and are not fully reversible over short time periods (<5 h).

## 4. Materials and Methods

### 4.1. Polymer Synthesis

PBzMA homopolymers were synthesized by atom transfer radical polymerization, adapting a procedure reported by Kharel et al. [31]. Copper (I) bromide, 1,1,4,7,10,10-hexamethyltriethylenetetramine (HMTETA), ethyl 2-bromoisobutyrate (EBr*i*B), and anisole were purchased from Sigma-Aldrich (St. Louis, MO, USA) and used as received. Copper (I) bromide was stored in an inert argon-atmosphere glove box. Benzyl methacrylate was purchased from Tokyo Chemical Industry (Tokyo, Japan) and purified via filtration through a neutral alumina column prior to use. A representative polymerization is described. A Schlenk flask outfitted with a magnetic stir bar was charged with benzyl methacrylate (33 g, 188 mmol), HMTETA (67 mg, 0.29 mmol), EBr*i*B (58 mg, 0.30 mmol), and anisole (95 mL). The reaction vessel was sealed with a rubber septum and the reaction mixture was sparged with argon gas for 20 min. The vessel was then placed under positive pressure of argon. A needle was used to puncture the septum and relieve pressure within the vessel. Under a constant flow of argon, the septum was removed, and copper (I) bromide (0.25 mg, 0.17 mmol) was added to the reaction mixture. The Schlenk flask was sealed and the argon pressure within the vessel increased to 5 atm. The reaction was allowed to proceed at 80 °C under constant stirring. After 12 h, the reaction was quenched by opening the reaction mixture to atmosphere and placing the Schlenk flask in liquid nitrogen. The reaction solution was precipitated into cooled methanol, redissolved in dichloromethane (DCM), and filtered through a neutral alumina column to remove the copper complex. The residue was dissolved in DCM, precipitated into cooled methanol, and dried at 100 °C under dynamic vacuum (<200 mTorr) to afford PBzMA as a white powder. The degree of polymerization for three homopolymer samples was varied by changing the reaction time and ratio of monomer to initiator.

The weight-average molecular weight (*M*_w_) and dispersity (*Ð*) of the synthesized homopolymers were obtained from size exclusion chromatography performed in tetrahydrofuran (THF) at room temperature on an Agilent 1260 Infinity System (Agilent Technologies, Santa Clara, CA, USA). A Wyatt Optilab T-rEX (Wyatt Technology, Santa Barbara, CA, USA) refractive index detector monitored the eluent (Figure S1). The *M*_w_ and *Ð* are summarized in Table 1 as determined from Zimm plots produced by a Wyatt Dawn Heleos II multiangle laser light scattering detector, using the value of 0.144 mL/g for the d*n*/d*c* of PBzMA in THF [31]. The synthesized polymers are designated PBzMA-*x*, where *x* indicates the *M*_w_ of PBzMA in kg/mol. The polymer purity was assessed via ^1^H NMR spectroscopy in CD_2_Cl_2_ (Figure S2) with a Bruker Avance III HD nanobay AX-400 spectrometer (Bruker, Billerica, MA, USA) with a shielded Ascend magnet and a SampleXpress autosampler.

### 4.2. Solution Preparation

The ionic liquid [BMP][TFSI] was purchased from IoLiTec GmbH (Heilbronn, Germany) and dried under a dynamic vacuum for 48 h. An ^1^H NMR spectrum of the ionic liquid in CDCl_3_ is shown in Figure S2. PBzMA and [BMP][TFSI] were combined gravimetrically and dissolved in THF as a co-solvent. The solutions were stirred until they achieved homogeneity and were then filtered using a 0.2 μm PTFE syringe filter. THF was evaporated under stirring during a nitrogen purge and the remaining solution was dried at 80 °C in an oven under a dynamic vacuum (<200 mTorr) for 12 h. Samples were stored in a vacuum desiccator prior to experiments.

### 4.3. Cloud Point Measurements

The PBzMA/[BMP][TFSI] solutions were flame-sealed in glass ampoules under reduced pressure (<130 mTorr) and were placed onto a temperature-controlled heating stage. Optical transmittance measurements were conducted by directing a HeNe laser (wavelength *λ*_0_ = 632.8 nm) through the sample and focusing the beam on a photodiode detector while the stage temperature was heated or cooled at ±1.0 °C/min. For varying ramp rate experiments, four ampoules were charged with a 10 wt% PBzMA-32 solution, flame-sealed, and subjected to temperature ramps at ±0.1, ±0.5, ±1.0, and ±5.0 °C/min. Labview software (National Instruments, Austin, TX, USA) automated the temperature control and data collection processes, recording the laser intensity every 5 s. The raw intensity values were averaged at each temperature and normalized against the maximum intensity throughout the temperature ramp, yielding a relative transmittance at each temperature. The cloud point temperature (*T*_CP_) is defined as the temperature when the transmittance drops to 80% of the value for the original homogenous solution.

## 5. Conclusions

The LCST-type phase behavior of PBzMA/[BMP][TFSI] concentrated solutions was explored for three molecular weights via optical transmittance. The phase separation temperatures demonstrate a strong dependence on concentration, decreasing as the incorporation of PBzMA increases, but are not influenced by molecular weight. The former trend is hypothesized to reflect a composition-dependent interaction parameter, while the latter is quite common in LCST systems. The critical compositions for these systems were shown to be shifted toward the polymer-rich regime of the phase diagram, in contrast to the solvent-rich regime anticipated by Flory-Huggins theory. These unusual characteristics add to the list of interesting solution phenomena exhibited by polymers in ILs. We also report the reversibility and thermal sensitivity of this phase transition to serve as a guide for polymer processing and thermo-responsive material design.

## Figures and Tables

**Table 1:** Polymer characteristics.

Sample	*M*_w_ (kDa) ^1^	*Ð* ^1^	*N* ^2^	*Φ*_c,th_ ^3^
PBzMA-32	32	1.20	90	11
PBzMA-63	63	1.15	177	8
PBzMA-76	76	1.18	213	7

^1^ *M*_w_ and *Ð* calculated via SEC-MALS in THF. ^2^ *N* is the volumetric degree of polymerization with respect to the molar volume of [BMP][TFSI], calculated using the room temperature densities of [BMP][TFSI] and PBzMA (1.40 g/cm^3^ and 1.18 g/cm^3^, respectively). ^3^ The theoretical critical composition, *Φ*_c,th_, in [BMP][TFSI] was estimated from Flory–Huggins theory (*Φ*_c,th_ ≈ *N*^−1/2^).

## Data Availability

Data is contained within the article and the Supplementary Materials.

## Supplementary Materials

The following are available online. Figure S1: Annotated ^1^H NMR spectrum of PBzMA-76 in CD_2_Cl_2_, Figure S2: Annotated ^1^H NMR spectrum of [BMP][TFSI] in CDCl_3_, Figure S3: Temperature dependence of relative transmittance for 40 wt% PBzMA-63 in [BMP][TFSI] with solution images.

## Sample Availability

Samples of the compounds are not available from the authors.

## References

[1] Ueki T., Watanabe M. (2008). Macromolecules in Ionic Liquids: Progress, Challenges, and Opportunities. Macromolecules.

[2] Ueki T., Watanabe M. (2012). Polymers in Ionic Liquids: Dawn of Neoteric Solvents and Innovative Materials. Bull. Chem. Soc. Jpn..

[3] Macfarlane D.R., Meakin P., Sun J., Amini N., Forsyth M. (1999). Pyrrolidinium Imides: A New Family of Molten Salts and Conductive Plastic Crystal Phases. J. Phys. Chem. B.

[4] Tang B., White S.P., Daniel Frisbie C., Lodge T.P. (2015). Synergistic Increase in Ionic Conductivity and Modulus of Triblock Copolymer Ion Gels. Macromolecules.

[5] Tokuda H., Ishii K., Abu Bin M., Susan H., Tsuzuki S., Hayamizu K., Watanabe M. (2006). Physicochemical Properties and Structures of Room-Temperature Ionic Liquids. 3. Variation of Cationic Structures. J. Phys. Chem. B.

[6] Ueki T., Yamaguchi A., Ito N., Kodama K., Sakamoto J., Ueno K., Kokubo H., Watanabe M. (2009). Photoisomerization-Induced Tunable LCST Phase Separation of Azobenzene-Containing Polymers in an Ionic Liquid. J. Polym. Sci. Part A.

[7] He Y., Boswell P.G., Bu P., Lodge T.P. (2007). Ion Gels by Self-Assembly of a Triblock Copolymer in an Ionic Liquid. J. Phys. Chem. B.

[8] Lodge T.P., Ueki T. (2016). Mechanically Tunable, Readily Processable Ion Gels by Self-Assembly of Block Copolymers in Ionic Liquids. Acc. Chem. Res..

[9] Tomé L.C., Mecerreyes D., Freire C.S., Paulo Rebelo L.N., Marrucho I.M. (2013). Pyrrolidinium-based polymeric ionic liquid materials: New perspectives for CO_2_ separation membranes. J. Membr. Sci..

[10] Gu Y., Cussler E.L., Lodge T.P. (2012). ABA-triblock copolymer ion gels for CO2 separation applications. J. Memb. Sci..

[11] Lodge T.P. (2008). A Unique Platform for Materials Design. Science.

[12] Ueki T. (2014). Stimuli-responsive polymers in ionic liquids. Polym. J..

[13] Choi J.H., Xie W., Gu Y., Daniel Frisbie C., Lodge T.P. (2015). Single Ion Conducting, Polymerized Ionic Liquid Triblock Copolymer Films: High Capacitance Electrolyte Gates for n-type Transistors. ACS Appl. Mater. Interfaces.

[14] Bai J., Lu H., Cao Y., Li X., Wang J. (2017). A novel ionic liquid polymer electrolyte for quasi-solid state lithium air batteries. RSC Adv..

[15] Ueki T., Usui R., Kitazawa Y., Lodge T.P., Watanabe M. (2015). Thermally Reversible Ion Gels with Photohealing Properties Based on Triblock Copolymer Self-Assembly. Macromolecules.

[16] Flory P.J. (1953). Principles of Polymer Chemistry.

[17] Lodge T.P., Hiemenz P.C. (2020). Polymer Chemistry.

[18] Tsuda R., Kodama K., Ueki T., Kokubo H., Imabayashi S.I., Watanabe M. (2008). LCST-type liquid-liquid phase separation behaviour of poly(ethylene oxide) derivatives in an ionic liquid. Chem. Commun..

[19] Kodama K., Tsuda R., Niitsuma K., Tamura T., Ueki T., Kokubo H., Watanabe M. (2011). Structural effects of polyethers and ionic liquids in their binary mixtures on lower critical solution temperature liquid-liquid phase separation. Polym. J..

[20] Lee H.-N., Lodge T.P. (2010). Lower Critical Solution Temperature (LCST) Phase Behavior of Poly(ethylene oxide) in Ionic Liquids. J. Phys. Chem. Lett..

[21] Lee H.N., Newell N., Bai Z., Lodge T.P. (2012). Unusual Lower Critical Solution Temperature Phase Behavior of Poly(ethylene oxide) in Ionic Liquids. Macromolecules.

[22] Costa L.T., Ribeiro M.C.C. (2006). Molecular dynamics simulation of polymer electrolytes based on poly(ethylene oxide) and ionic liquids. I. Structural properties. J. Chem. Phys..

[23] Costa L.T., Ribeiro M.C.C. (2007). Molecular dynamics simulation of polymer electrolytes based on poly(ethylene oxide) and ionic liquids. II. Dynamical properties. J. Chem. Phys..

[24] Lee H.-N., Lodge T.P. (2011). Poly(n-butyl methacrylate) in Ionic Liquids with Tunable Lower Critical Solution Temperatures (LCST). J. Phys. Chem. B.

[25] Hoarfrost M.L., He Y., Lodge T.P. (2013). Lower Critical Solution Temperature Phase Behavior of Poly(n-butyl methacrylate) in Ionic Liquid Mixtures. Macromolecules.

[26] Ueki T., Watanabe M. (2007). Lower Critical Solution Temperature Behavior of Linear Polymers in Ionic Liquids and the Corresponding Volume Phase Transition of Polymer Gels. Langmuir.

[27] Ueki T., Karino T., Kobayashi Y., Shibayama M., Watanabe M. (2007). Difference in Lower Critical Solution Temperature Behavior between Random Copolymers and a Homopolymer Having Solvatophilic and Solvatophobic Structures in an Ionic Liquid †. J. Phys. Chem. B.

[28] Ueki T., Arai A.A., Kodama K., Kaino S., Takada N., Morita T., Nishikawa K., Watanabe M. (2009). Thermodynamic Study of Phase Transitions of Poly(benzyl methacrylate) in Ionic Liquid Solvents. Pure Appl. Chem..

[29] Fujii K., Ueki T., Niitsuma K., Matsunaga T., Watanabe M., Shibayama M. (2011). Structural aspects of the LCST phase behavior of poly(benzyl methacrylate) in room-temperature ionic liquid. Polymer.

[30] Fujii K., Ueki T., Hashimoto K., Kobayashi Y., Kitazawa Y., Hirosawa K., Matsugami M., Ohara K., Watanabe M., Shibayama M. (2017). Microscopic Structure of Solvated Poly(benzyl methacrylate) in an Imidazolium-Based Ionic Liquid: High-Energy X-ray Total Scattering and All-Atom MD Simulation Study. Macromolecules.

[31] Kharel A., Hall C., Ernoch P.C., Stepanek P., Lodge T.P. (2020). Dilute Solution Properties of Poly(benzyl methacrylate) in Ionic Liquids. Macromolecules.

[32] Kaiser Custodio K.S., Claudio G.C., Nellas R.B. (2020). Structural Dynamics of Neighboring Water Molecules of N-Isopropylacrylamide Pentamer. ACS Omega.

[33] Kobayashi Y., Kitazawa Y., Hashimoto K., Ueki T., Kokubo H., Watanabe M. (2017). Thermosensitive Phase Separation Behavior of Poly(benzyl methacrylate)/Solvate Ionic Liquid Solutions. Langmuir.

[34] Hashimoto K., Kobayashi Y., Kokubo H., Ueki T., Ohara K., Fujii K., Watanabe M. (2019). Solvation Structure of Poly(benzyl methacrylate) in a Solvate Ionic Liquid: Preferential Solvation of Li-Glyme Complex Cation. J. Phys. Chem. B.

[35] Ventura S.P.M., Gonçalves A.M.M., Sintra T., Pereira J.L., Gonçalves F., Coutinho J.A.P. (2013). Designing ionic liquids: The chemical structure role in the toxicity. Ecotoxicology.

[36] Halperin A., Krçger M., Winnik F.M. (2015). Polymer Phase Diagrams Poly(N-isopropylacrylamide) Phase Diagrams: Fifty Years of Research. Angew. Chem. Int. Ed..

[37] Rackaitis M., Strawhecker K., Manias E. (2002). Water-soluble polymers with tunable temperature sensitivity: Solution behavior. J. Polym. Sci. Part B Polym. Phys..

[38] White R.P., Lipson J.E.G. (2013). Origins of Unusual Phase Behavior in Polymer/Ionic Liquid Solutions. Macromolecules.

[39] Gennes P.G. (2009). Special Features of Water Soluble Polymers. Pure Appl. Chem..

[40] Schäfer-Soenen H., Moerkerke R., Berghmans H., Koningsveld R., Dušek K., Šolc K. (1997). Zero and off-zero critical concentrations in systems containing polydisperse polymers† with very high molar masses. 2. The system water-poly(vinyl methyl ether). Macromolecules.

