# Supplementary material for: Unusual Lower Critical Solution Temperature Phase Behavior of Poly(benzyl methacrylate) in a Pyrrolidinium-Based Ionic Liquid
Source: Molecules. 2021 Aug 11;26(16):4850. doi: 10.3390/molecules26164850 (PMC8399651; doi:10.3390/molecules26164850)
Supplement: Supplementary file 1 [file molecules-26-04850-s001.zip › molecules-1329440-supplementary.pdf]

SUPPLEMENTARY MATERIALS FOR

Unusual Lower Critical Solution Temperature Phase Behavior of Poly(benzyl methacrylate) in a Pyrrolidinium-Based Ionic Liquid

Brian R. Carrick[†‡], Claire L. Seitzinger[†], and Timothy P. Lodge[†‡*]

† Department of Chemistry, University of Minnesota, Minneapolis, Minnesota 55455-0431, United States

‡ Department of Chemical Engineering and Materials Science, University of Minnesota, Minneapolis, Minnesota 55455-0431, United States

* Corresponding Author: lodge@umn.edu

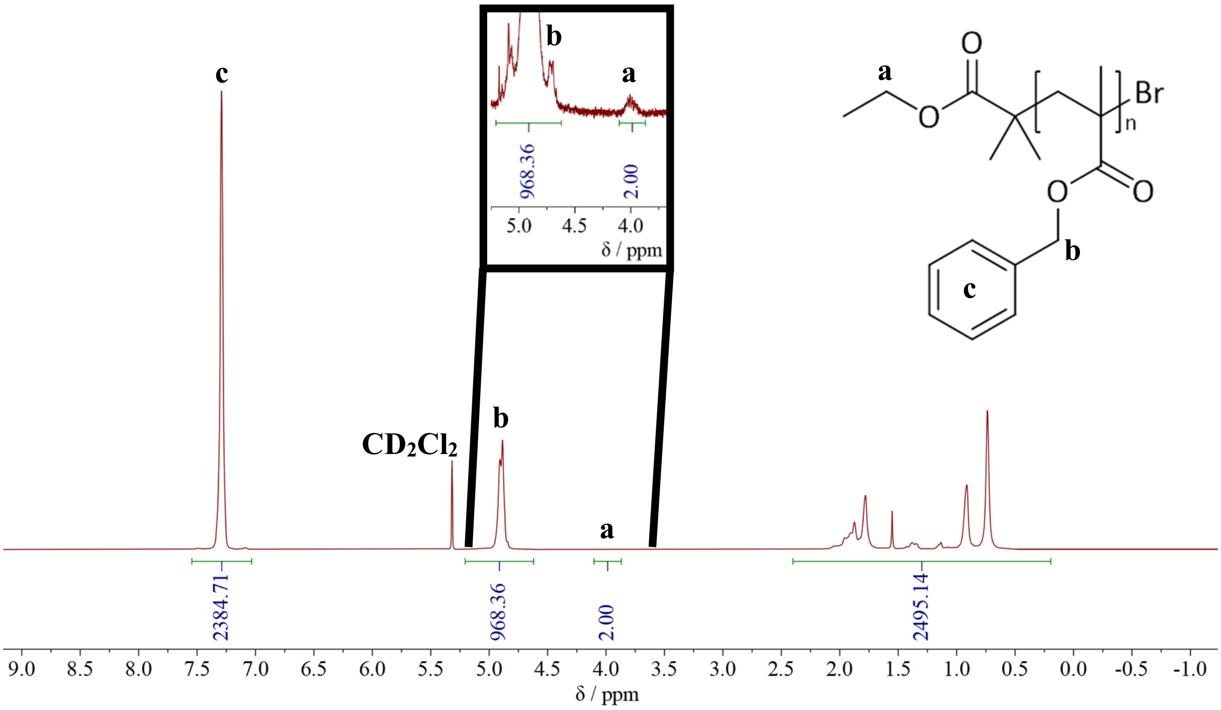

Figure S1: Annotated ^{1}H NMR spectrum of PBzMA-76 in CD_2Cl_2.

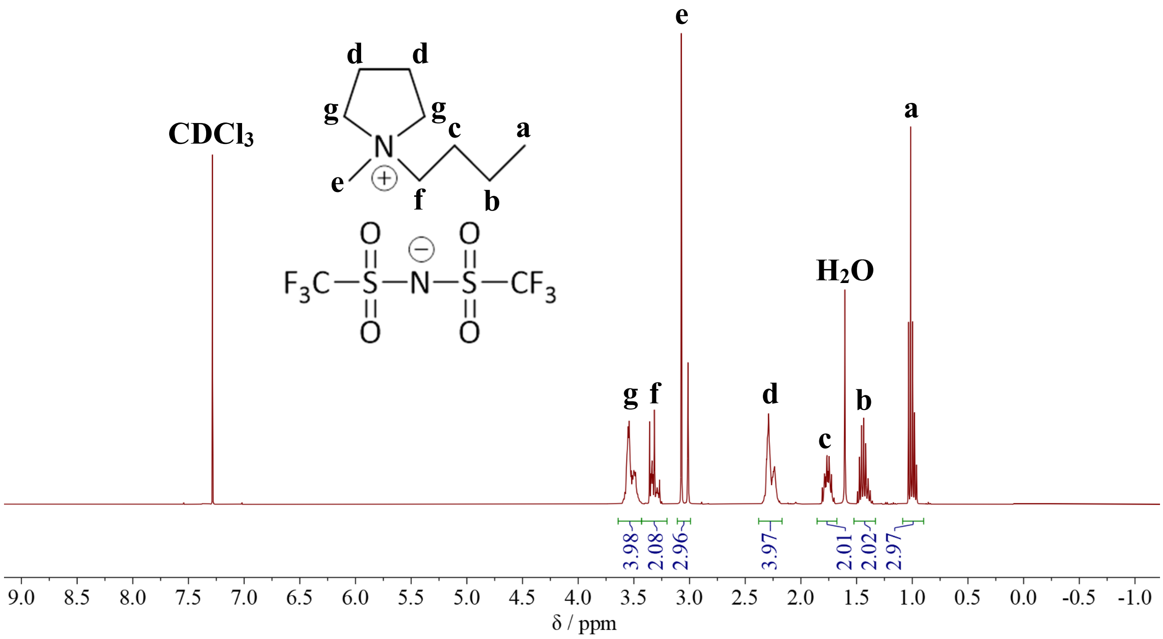

Figure S2: Annotated ^{1}H NMR spectrum of [BMP][TFSI] in $CDCl_3$.

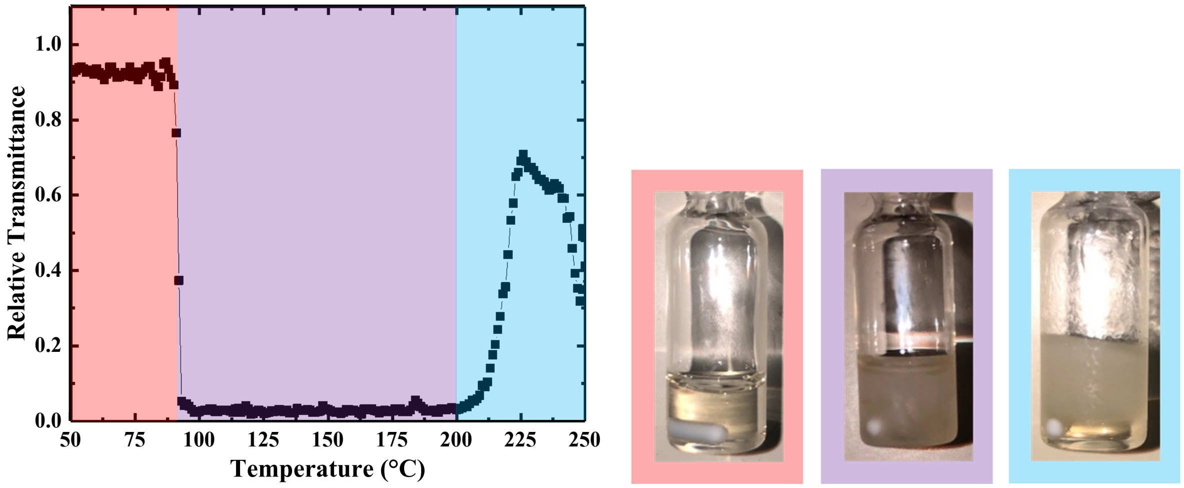

Figure S3: Temperature dependence of relative transmittance for 40 wt% PBzMA-63 in [BMP][TFSI] at a heating rate of 1 °C/min. The images represent the solutions at stages characterized by the corresponding colored regimes on the graph ordered from left to right: (red) a homogenous, single phase solution, (purple) a biphasic mixture during phase separation, and (blue) PBzMA-rich biphasic top layer with a [BMP][TFSI]-rich bottom layer. The solid line serves as a guide to the eye.
